# HEXIM1 Induces Differentiation of Human Pluripotent Stem Cells

**DOI:** 10.1371/journal.pone.0072823

**Published:** 2013-08-20

**Authors:** Vanessa Ding, Qiao Jing Lew, Kai Ling Chu, Subaashini Natarajan, Vikneswari Rajasegaran, Meera Gurumurthy, Andre B. H. Choo, Sheng-Hao Chao

**Affiliations:** 1 Stem Cell Group, Bioprocessing Technology Institute, A*STAR (Agency for Science, Technology and Research), Singapore, Singapore; 2 Expression Engineering Group, Bioprocessing Technology Institute, A*STAR (Agency for Science, Technology and Research), Singapore, Singapore; 3 Department of Bioengineering, Faculty of Engineering, National University of Singapore, Singapore, Singapore; 4 Department of Microbiology, National University of Singapore, Singapore, Singapore; Baylor College of Medicine, United States of America

## Abstract

Hexamethylene bisacetamide inducible protein 1 (HEXIM1) is best known as the inhibitor of positive transcription elongation factor b (P-TEFb), which is composed of cyclin-dependent kinase 9 (CDK9)/cyclin T1. P-TEFb is an essential regulator for the transcriptional elongation by RNA polymerase II. A genome-wide study using human embryonic stem cells shows that most mRNA synthesis is regulated at the stage of transcription elongation, suggesting a possible role for P-TEFb/HEXIM1 in the gene regulation of stem cells. In this report, we detected a marked increase in HEXIM1 protein levels in the differentiated human pluripotent stem cells (hPSCs) induced by LY294002 treatment. Since no changes in CDK9 and cyclin T1 were observed in the LY294002-treated cells, increased levels of HEXIM1 might lead to inhibition of P-TEFb activity. However, treatment with a potent P-TEFb inhibiting compound, flavopiridol, failed to induce hPSC differentiation, ruling out the possible requirement for P-TEFb kinase activity in hPSC differentiation. Conversely, differentiation was observed when hPSCs were incubated with hexamethylene bisacetamide, a HEXIM1 inducing reagent. The involvement of HEXIM1 in the regulation of hPSCs was further supported when overexpression of HEXIM1 concomitantly induced hPSC differentiation. Collectively, our study demonstrates a novel role of HEXIM1 in regulating hPSC fate through a P-TEFb-independent pathway.

## Introduction

Pluripotent stem cells (PSCs) such as human embryonic stem cells (hESCs) [1,2] and induced pluripotent stem (iPS) cells [3,4] have enormous potential for regenerative medicine because of their ability to proliferate indefinitely and to differentiate into all three germ layers under appropriate conditions. These include lineage-specific cell types, such as cardiomyocytes [5–7], insulin-producing cells [8,9], and neural-like cells [10–12]. Concurrently, significant efforts have been spent focusing on the mechanisms and pathways regulating hPSC self-renewal and directed differentiation.

Positive transcription elongation factor b (P-TEFb), a protein complex composed of cyclin-dependent kinase 9 (CDK9) and a cyclin partner, with cyclin T1 being the predominant CDK9-associated cyclin, plays a crucial role in the regulation of RNA polymerase II (Pol II) transcription elongation [13–15]. Treatment of P-TEFb inhibiting compounds, such as flavopiridol, blocks RNA Pol II at the pre-elongation phase and inhibits most of mRNA synthesis in cells [16–19]. This observation clearly demonstrates that transcription of most cellular genes is regulated at the elongation stage, which is controlled by P-TEFb. Genome-wide analyses of *Drosophila* and hESCs reveal that many genes required for differentiation and development are regulated at the stage of transcription elongation, affirming the importance of P-TEFb in regulation of gene expression [20–23].

In cells, the activity of P-TEFb is tightly regulated by its inhibitor, hexamethylene bisacetamide inducible protein 1 (HEXIM1). Two P-TEFb protein complexes are found in cells. The small, active complex consists of CDK9 and cyclin T1. The large, inactive P-TEFb complex is formed when the small P-TEFb complex associates with HEXIM1 and a small nuclear RNA (snRNA) [24–27]. HEXIM1 was first identified from vascular smooth muscle cells treated with hexamethylene bisacetamide (HMBA), a proliferation-inhibiting and differentiation-inducing compound. Treatment of HMBA led to increases in both mRNA and protein levels of HEXIM1 [28–30]. HEXIM1 functions as a P-TEFb inhibitor and the mechanism of P-TEFb inhibition by HEXIM1 has been revealed. HEXIM1 first forms a homodimer via its C-terminus, and then the homodimer associates with 7SK snRNA, resulting in a conformational change and exposing its C-terminal domain for CDK9/cyclin T1 binding. Once binding to HEXIM1-7SK snRNA complexes, the kinase activity of P-TEFb is inhibited [17,25,31]. About 50% of P-TEFb is found to associate with HEXIM1 in cells, suggesting the importance of HEXIM1 in the regulation of P-TEFb [25]. Besides P-TEFb, other HEXIM1 binding proteins were identified, including MyoD, histone deacetylases, importin alpha, HDM2, nucleophosmin (NPM), p53, estrogen receptor alpha (ERα), NF-κB, and glucocorticoid receptor (GR) [32–39]. Some of the HEXIM1 binding proteins, such as HDM2 and NPM, regulate P-TEFb activity through the modulation of HEXIM1 proteins [34,35]. On the other hand, HEXIM1 can affect the functions of its binding proteins in the P-TEFb-dependent (such as ERα) or -independent (such as GR) manners [36,38].

In this study, we show that treatment with HMBA induces hPSC differentiation and increases the protein levels of HEXIM1. However, no signs of differentiation were detected when hPSCs were incubated with a potent P-TEFb-inhibiting compound, flavopiridol. Overexpression of HEXIM1 induced differentiation even when these cells were cultured in pluripotent conditions. Taking together, our results demonstrate a novel role of HEXIM1 in the regulation of hPSC pluripotency through a P-TEFb-independent mechanism. 

## Materials and Methods

### Cell culture and western blotting

The human embryonic stem cell lines, HES-2 and HES-3, were obtained from ES Cell International and cultured on Matrigel [Becton, Dickinson and Company (BD)] in conditioned medium (CM) containing fibroblast growth factor-2 (FGF-2) (Invitrogen). The CM was obtained from immortalized mouse feeders as previously described [40,41]. Induced pluripotent stem cells (iPS-IMR90), derived from lung fibroblasts, were kindly provided by Dr. James Thomson [4]. The iPS cells were cultured as per the hESC culture, with the exception that 100 ng/ml of FGF-2 was supplemented to the CM [4]. To induce differentiation, cells were harvested as clumps and cultured as embryoid bodies (EB) for a week in EB medium [80% KO-DMEM, 20% fetal bovine serum, 1% MEM non-essential amino acids, 1mM L-glutamine, 1% penicillin-streptomycin, (Invitrogen) and 0.1 mM β-mercaptoethanol (Sigma-Aldrich)] on non-adherent suspension culture plates (Life Sciences). The EBs were then dissociated with trypsin, and plated on gelatinized tissue culture dishes in EB medium for another 2 weeks prior analysis [42]. Western blotting was performed according to standard protocols. Anti-actin, -CDK9, -cyclin T1, and -OCT3/4 antibodies were purchased from Santa Cruz Biotechnology. The anti-HEXIM1 antibody was kindly provided by Dr. Olivier Bensaude [25].

### Compound treatment

HES-3 cells were treated with 20 µM LY294002 (Cell Singaling Technology) for 7 population doublings (PDs, 1 PD = ~24hrs) in the presence of FGF-2 as previously described [43]. 0.2% dimethylsulfoxide (DMSO) was used as vehicle control. Treated cells were dissociated using trypsin and lysed for western blotting. HMBA and flavopiridol were purchased from Sigma and dissolved in ethanol and DMSO, respectively. To examine the influence of HMBA and flavopiridol on the expression of pluripotent markers, cells were seeded onto 6-well dishes and allowed to adhere for 24 hrs, followed by HMBA and flavopiridol incubation at the indicated concentrations. The final concentrations of DMSO and ethanol in the cell culture were kept at 0.2 and 1%, respective. Cells were then further cultured for 7 PDs and harvested for flow cytometry.

### Fluorescence-activated cell sorting (FACS) analysis

To determine the effects of flavopiridol and HMBA on the pluripotency of hPSCs, the compound-treated HES-2, HES-3, and iPS cells were trypsinized and incubated with an indicated antibody, including anti-OCT3/4, anti-Tra-1-60 (Millipore), and anti-PODXL [42]. Cells were then incubated with a FITC-conjugated secondary antibody (Dako) and analyzed on a FACSCalibur™ (BD). The FlowJo software (Tree Star, Inc.) was utilized for data processing.

### Generation of HEXIM-1-overexpressing cell line

The coding region of HEXIM1 was amplified by polymerase chain reaction (PCR) using the pcDNA6-HEXIM1 vector as the template [35,44]. The amplified DNA fragment was subcloned into a pCHEF-1 vector to generate the HEXIM1 expression plasmid, pCHEF-1-HEXIM1, in which the expression of HEXIM1 was driven by Chinese hamster elongation factor-1alpha (CHEF-1) promoter [35,44]. HES-3 cells were cultured in a 12-well plate and transfected with 2.4µg/well of pCHEF-1-HEXIM1 using Lipofectamine 2000 according to manufacturer’s instruction (Invitrogen). Two days post-transfection, cells were subject to 10 µg/ml of blasticidin antibiotic (Invitrogen) selection daily for 5 PDs. The blasticidin resistant cells were either harvested for western blotting to confirm HEXIM1 overexpression or were cultured with continuous blasticidin selection and scaled up over 3 passages before being harvested for cell sorting.

The HEXIM1-overexpressing cells were harvested using TrypLE™ Express (Invitrogen). Surface antigens were labeled with an anti-Tra-1-60 antibody, followed by incubation with a FITC-conjugated fluorescent secondary antibody. Prior to sorting, the cells were filtered through cell strainer caps (40µm mesh) (BD) to obtain a single cell suspension (approximately 5×10^6^ cells/ml). The stained cells were analyzed and sorted into two distinct cell populations, Tra-1-60 positive and Tra-1-60 negative, on a fluorescence-activated cell sorter FACSAriaII (BD) using FACSDiva software (BD). Dead cells were identified and eliminated by propidium iodide staining. The Tra-1-60 positive populations were determined according to fluorescence in the green channel as compared with a negative control which lacked the primary antibody. The two sorted cell populations were then individually analyzed by quantitative real-time polymerase chain reaction (QRT-PCR) to examine the expression of HEXIM1 and marker genes of three germ layers.

### QRT-PCR

Total RNA was extracted from the cells using the Nucleospin RNA II kit (Macherey-Nagel) according to the manufacturer’s protocol. Reverse transcription was carried out with 0.16 µg of total RNA (for HMBA-treated cells) and 1 µg of total RNA (for HEXIM1-transfected cells and LY294002-treated cells) using M-MLV Reverse Transcriptase (Promega). QRT-PCR analysis was performed using an ABI PRISM 7500 Sequence Detection System and SYBR green PCR Master Mix (Applied Biosystems). Primers used are listed in Table S1. Fold induction was calculated relative to expression of 18S rRNA or glyceraldehyde 3-phosphate dehydrogenase (GAPDH) using the ΔΔCt method [45].

## Results

### Expression of CDK9, cyclin T1, and HEXIM1 in the pluripotent and differentiated hPSCs

**Figure 1:**
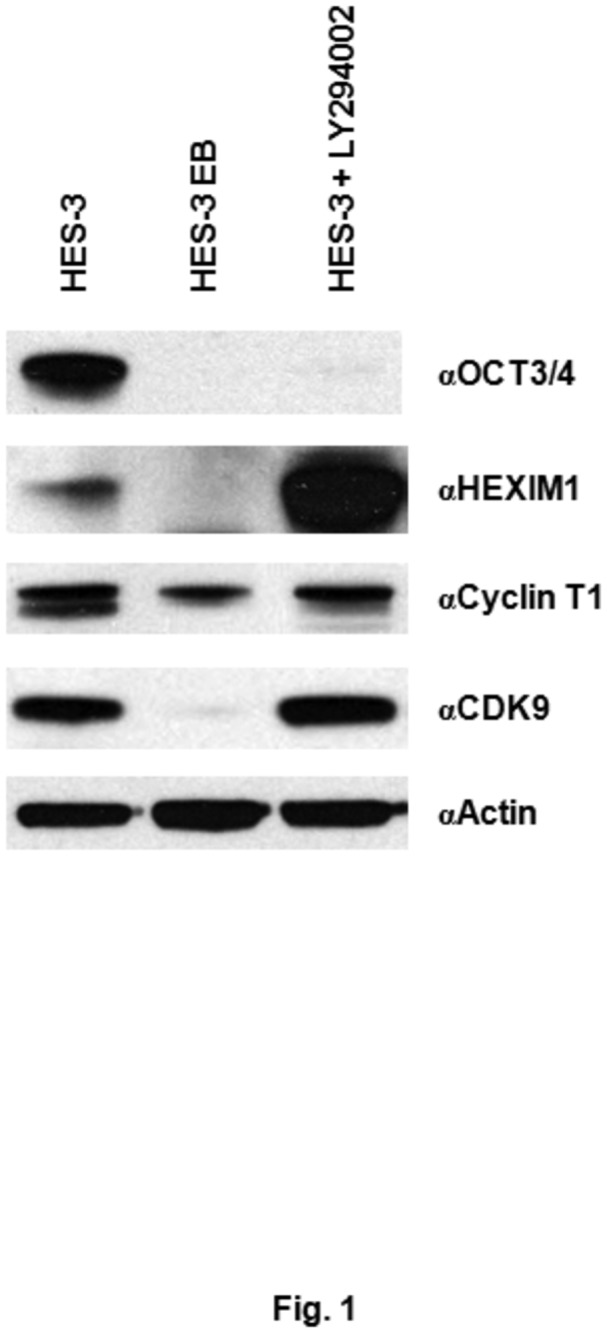
Differential protein expression of CDK9, cyclin T1, and HEXIM1 in pluripotent and differentiated hPSCs. Cell lysates prepared from HES-3, HES-3-derived EB, and LY294002-treated HES-3 cells were analyzed by western blotting. OCT3/4 is a pluripotent marker and actin is used as a loading control.

It has been shown that many cellular genes required for hESC differentiation are regulated at the stage of transcription elongation, which is controlled by P-TEFb/HEXIM1 [20]. To investigate the possible involvement of P-TEFb/HEXIM1 in hPSC pluripotency and differentiation, we examined the protein levels of HEXIM1, cyclin T1, and CDK9 in HES-3 hPSC, HES-3-derived EBs, and LY294002-treated HES-3 cells. LY294002, a phosphoinositide 3-kinases inhibitor and a differentiation-inducing compound [46], was used to differentiate HES-3 cells, as shown in our previous study [43]. As shown in Figure 1, expression of OCT3/4, a pluripotent marker of stem cells, was only detected in HES-3 but not in the other two differentiated cells [47]. Compared to HES-3 cells, decreased levels of HEXIM1, cyclin T1, and CDK9 proteins were observed in HES-3 EBs (Figure 1). A significant higher level of HEXIM1 was detected in LY294002-treated-HES-3 cells than that in HES-3 cells (Figure 1). However, similar expression levels of cyclin T1 and CDK9 were detected between HES-3 and LY294002-treated-HES-3 cells (Figure 1). We further examined the effect of LY294002 on the transcription of HEXIM1 by QRT-PCR. Treatment with LY294002 only resulted in a 20% increase in the HEXIM1 mRNA, suggesting that LY294002 might regulate HEXIM1 at translational stage, and not at transcriptional level (data not shown).

### Inhibition of P-TEFb does not induce hPSC differentiation

**Figure 2:**
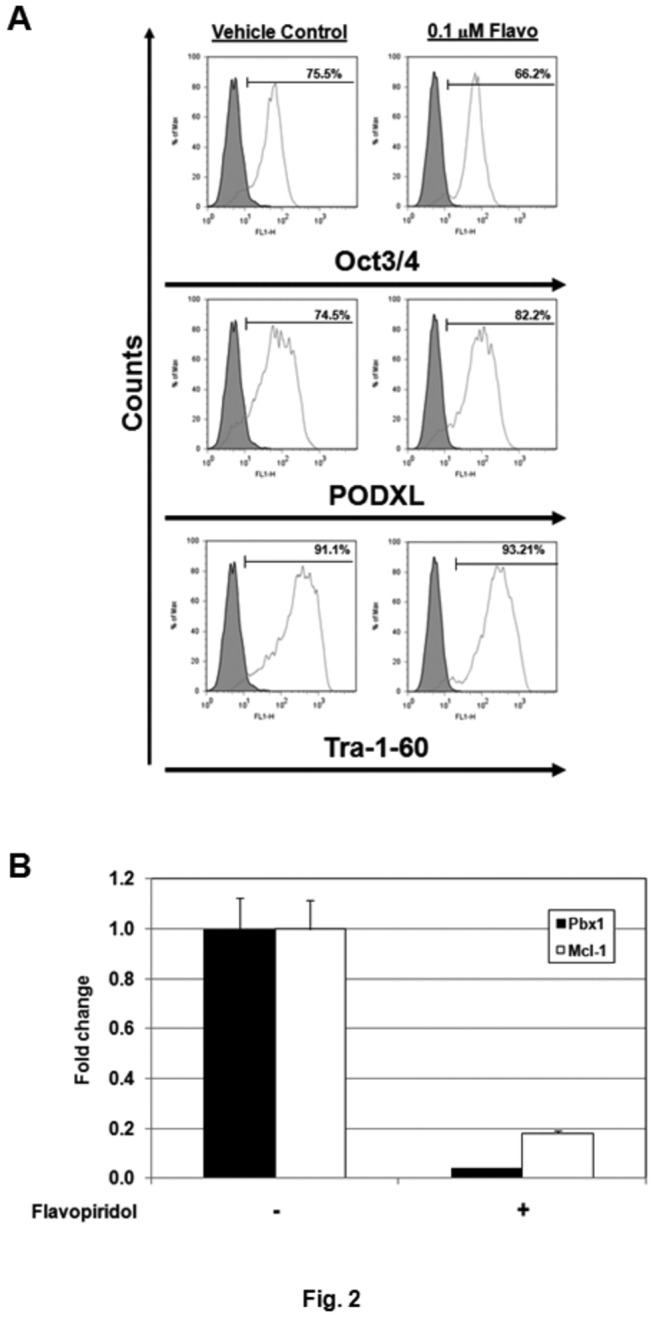
Treatment of flavopiridol does not induce differentiation of hPSC. (A) HES-3 cells were incubated with 0.1 µM flavopiridol for 7 PDs and the expression of pluripotent markers, OCT3/4, PODXL, Tra-1-60, were measured by flow cytometry. The shaded histograms represent staining with the negative control and open histograms represent staining with anti-OCT3/4, -PODXL, and -Tra-1-60 antibodies, respectively. Cells treated with 0.2% DMSO were used as the vehicle control for flavopiridol treatment. (B) HES-3 cells were incubated with 0.1 µM flavopiridol overnight and the expression of P-TEFb-dependent genes, Pbx1 and Mcl-1, was examined by QRT-PCR.

Decreases in cyclin T1/CDK9 in HES-3 EBs and increases in HEXIM1, a P-TEFb inhibitor, in LY294002-treated HES-3 cells suggested that lower kinase activity of P-TEFb might be required for hPSC differentiation (Figure 1). To assess the possible involvement of P-TEFb, HES-3 cells were incubated with flavopiridol, a selective and potent P-TEFb inhibiting compound with *K*_*i*_ (inhibitor constant) below 3 nM [16,18]. The expression of pluripotent markers for hPSCs, including OCT3/4, PODXL, and Tra-1-60, were analyzed by flow cytometry. No signs of differentiation were detected when cells were incubated with 0.1 µM or lower concentrations of flavopiridol (Figure 2A and data not shown). At higher concentrations (0.3 µM), flavopiridol was found to be toxic to cells leading to significant cell death (data not shown). This result was expected since it had been shown that 60-70% of total mRNA synthesis was inhibited in HeLa cells when treated with 0.3 µM flavopiridol for only one hour [18]. Massive cell death were usually observed when cells were treated with 0.3-1 µM flavopiridol, while incubation with 0.1 µM or lower concentrations of flavopiridol exhibited little or no cytotoxic effects [18,48]. Inhibition of P-TEFb-dependent genes, Pbx1 and Mcl-1 [34], was detected in HES-3 cells treated with 0.1 µM flavopiridol, indicating effective inhibition of P-TEFb activity under this condition (Figure 2B). Taken together, these results suggest that the kinase activity of P-TEFb is essential for viability of hPSCs but may not be a critical factor for inducing hPSC differentiation.

### Induction of hPSC differentiation by HMBA

**Figure 3:**
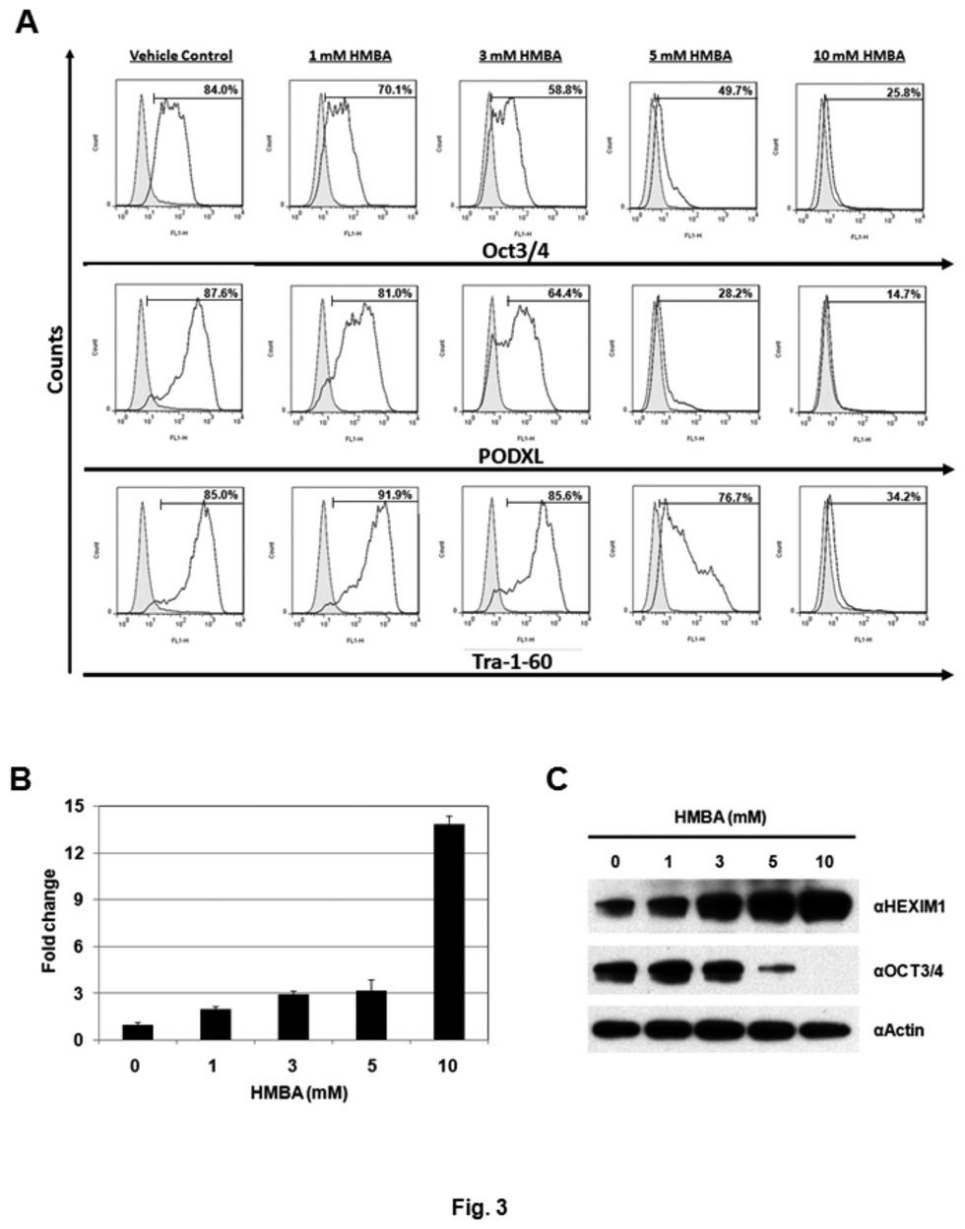
Treatment of HMBA increases expression of HEXIM1 and induces differentiation in HES-3 cells. (A) HES-3 cells were incubated with 1, 3, 5, or 10 mM HMBA for 7 PDs, followed by FACS analysis. Percentages of cells expressing pluripotent markers, including OCT3/4, PODXL, and Tra-1-60, were indicated (i.e. open histograms). Cells treated with 1% ethanol were used as the vehicle control. (B) HEXIM1 mRNA levels of the HMBA-treated HES-3 cells were measured by QRT-PCR. (C) HEXIM1 and OCT3/4 protein levels of the HMBA-treated HES-3 cells were examined by western blotting. Actin was used as a loading control.

Since P-TEFb activity may not be required for differentiation of hPSCs (Figure 2), it is possible that HEXIM1 may exert its own effects through a P-TEFb-independent mechanism. To induce the expression of HEXIM1, we treated HES-3 cells with HMBA, a HEXIM1 inducing compound [37], and examined its effects on the cells. No significant influence on differentiation of HES-3 cells was observed when cells were treated with 1 or 3 mM HMBA (Figure 3A). However, when the concentrations of HMBA were increased to 5 and 10 mM HMBA, significant decreases in the expression of pluripotent markers, including OCT3/4, PODXL, and Tra-1-60, were detected (Figure 3A). To confirm the action of HMBA, we examined the HEXIM1 levels in the HMBA-treated cells by QRT-PCR and western blotting. Gradient increases in HEXIM1 mRNA and protein levels were detected as the concentrations of HMBA increased (Figure 3B and C). Although the elevated expression of HEXIM1 was detected at 1 and 3 mM HMBA treatment, no signs of differentiation were observed (Figure 3). Decreases in OCT3/4 proteins were only detected when 5 mM or higher amounts of HMBA were used (Figure 3C). Changes in morphology of hPSCs were only observed when cells were incubated with high doses of HMBA. Cell treated with 5 mM of HMBA displayed a more differentiated morphology, evidenced by the appearance of the cystic-like regions (Figure S1).

To further assess if the effect of HMBA on hPSC differentiation was cell-specific, we incubated two other hPSC lines, HES-2 and iPS-IMR90, with HMBA. As shown in Figure S2A, the effect of treatment with 5 and 10 mM HMBA resulted in differentiation of HES-2 and iPS-IMR90 cells comparable to HES-3 cells. Up-regulation of HEXIM1 and down-regulation of OCT3/4 proteins in the HMBA-treated cells were also confirmed by western blot analyses (Figure S2B).

### HMBA induces lineage-specific differentiation of hPSC

**Figure 4:**
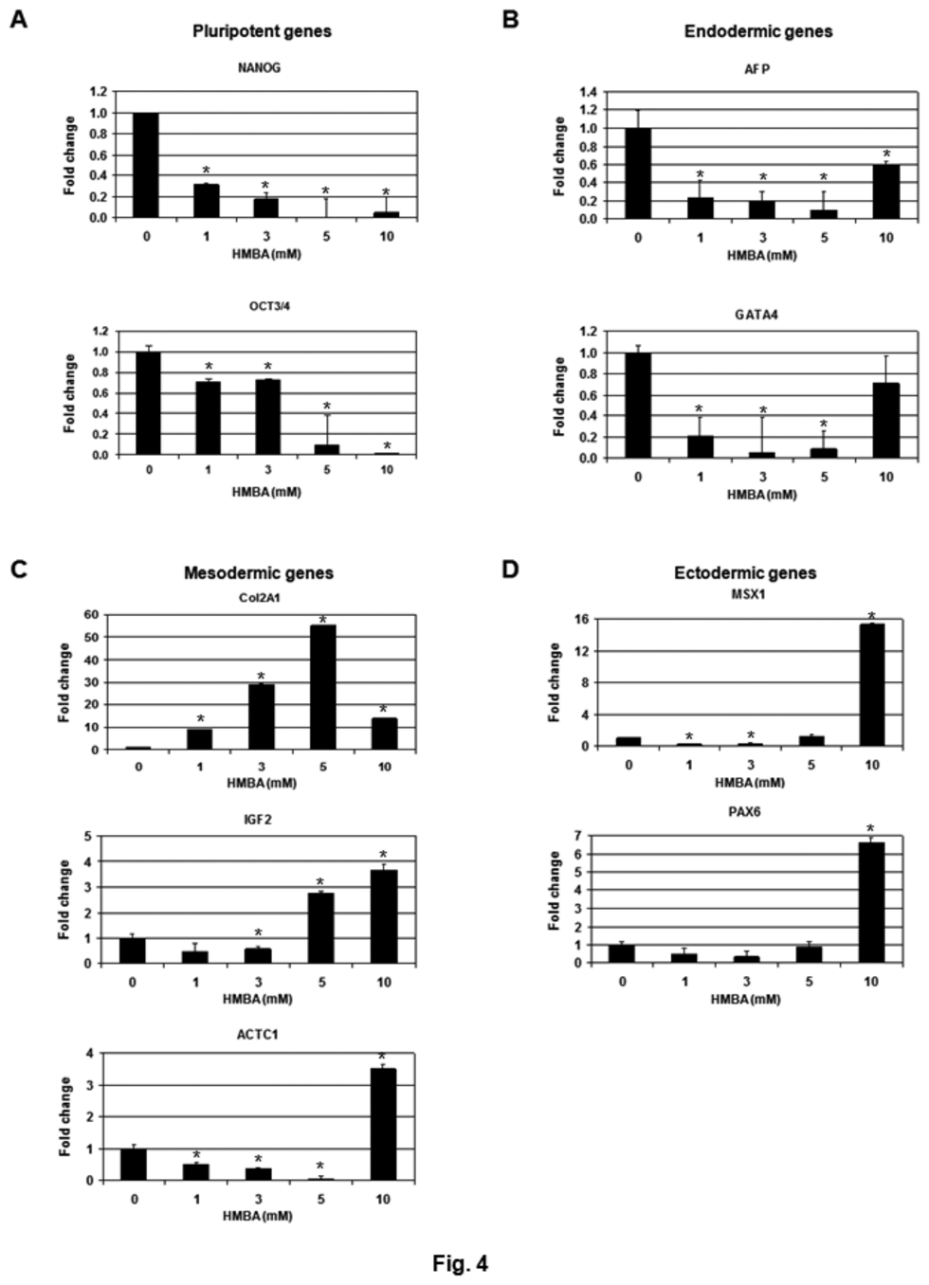
Treatment of HMBA differentiates hPSCs into mesoderm-ectoderm lineages. HES-3 cells were incubated with 1, 3, 5 or 10 mM HMBA for 7 PDs. RNAs prepared from the treated cells were analyzed by QRT-PCR to determine the expression of (A) pluripotent (NANOG and OCT3/4), (B) endodermal (AFP and GATA4), (C) mesodermal (Col2A1, IGF2, and ACTC1), and (D) ectodermal genes (MSX1, PAX6, and SOX1). Results were reproducible, and data from one experiment are presented. Error bars indicate the standard deviations between triplicates. *P<0.05 versus control.

Lineage commitment of the HMBA-induced hPSC differentiation was investigated next. As shown in Figure 4, HES-3 cells were treated with HMBA at the indicated concentrations and the expression of specific gene markers from each germ layer was analyzed by QRT-PCR. The action of HMBA was first confirmed by the repression of pluripotent genes, NANOG and OCT3/4 (Figure 4A). NANOG and OCT3/4 were completely repressed when cells were treated with 5 or 10 mM HMBA (Figure 4A). HMBA treatment did not up-regulate the expression of endodermal markers, alphafoetoprotein (AFP) or GATA4 (Figure 4B) [49,50]. However, up-regulation of mesodermal genes, including type II collagen (Col2A1), insulin-like growth factor 2 (IGF2), and α-cardiac muscle actin (ACTC1), was detected under HMBA treatment [51–53]. Col2A1 was most sensitive to HMBA. A 10-fold increase in Col2A1 mRNA was observed at 1 mM HMBA and the activation of Col2A1 reached the highest level (>50-fold) when treated with 5 mM HMBA (Figure 4C). Induction of IGF2 and ACTC1 was only detected when cells were incubated with 5 or 10 mM HMBA (Figure 4C). High concentrations of HMBA (i.e. 10 mM) also stimulated ectodermal differentiation as indicated by activation of msh homeobox 1 (MS X1), paired box 6 (PAX6), and SRY (sex determining region Y)-box 1 (SOX1), three ectodermal markers [54–56]. Collectively, our results demonstrate that HMBA directs hPSCs differentiating towards the mesoderm-ectoderm lineage.

The effects of LY294002 on lineage commitment in HES-3 cells was also investigated and similar results were observed. Activity of LY294002 was first confirmed by the repression of pluripotent genes (Figure S3A). Treatment with LY294002 potently inhibited endodermal marker genes (Figure S3B) but exhibited diverse effects of mesodermal and ectodermal markers (Figure S3C and D). We noticed that both HMBA and LY294002 treatments failed to induce endodermal differentiation in our study.

### Overexpression of HEXIM1 induces hPSC differentiation

Results from the treatment of HMBA suggested a possible involvement of HEXIM1 in hPSC differentiation. To investigate if HEXIM1 up-regulation had a causative role in hPSC differentiation, we overexpressed HEXIM1 in HES-3 cells using a HEXIM1 expression plasmid.

**Figure 5:**
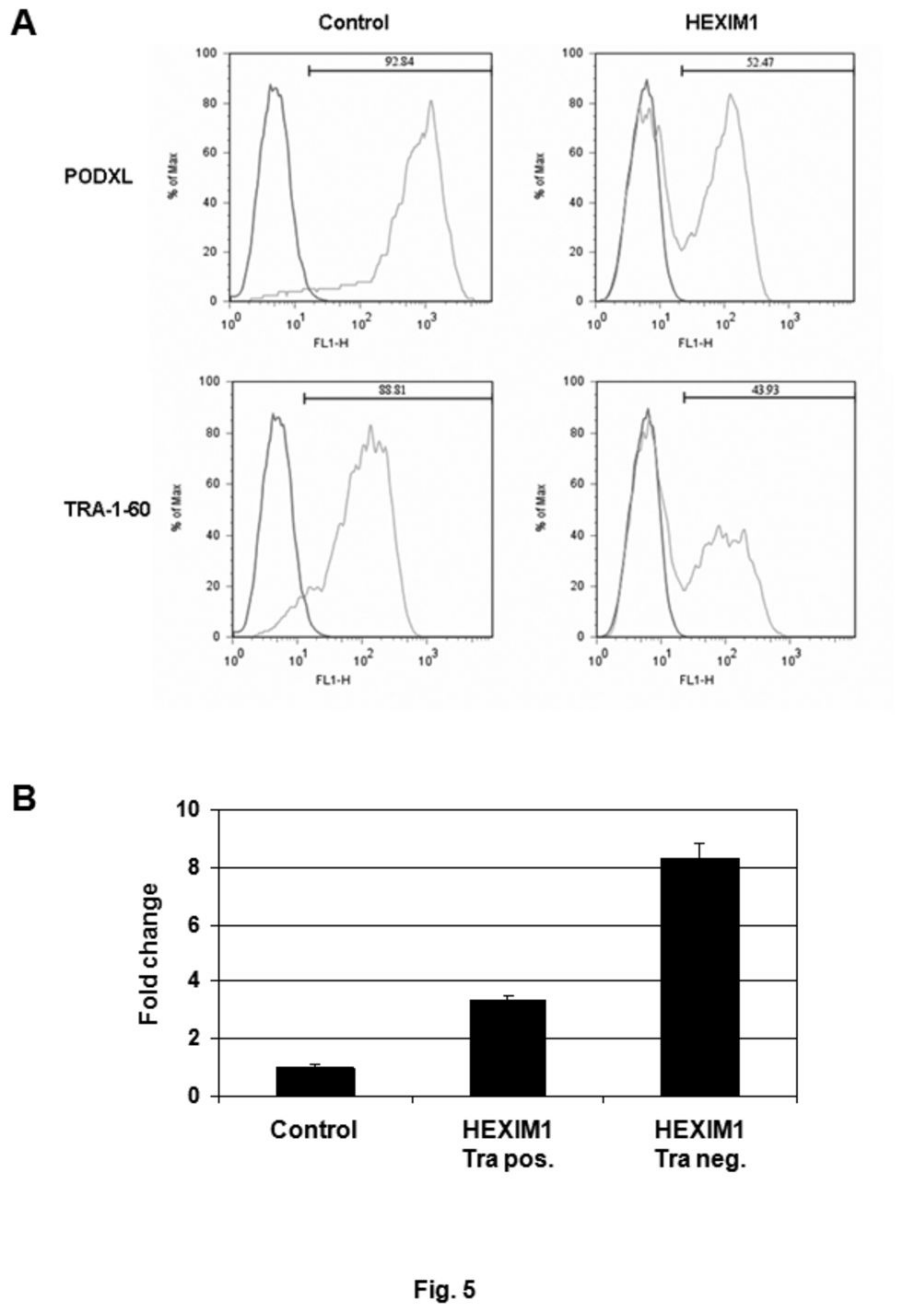
Overexpression of HEXIM1 induces differentiation of hPSCs. (A) HES-3 cells were transiently transfected with a HEXIM1 expression plasmid, and selected using blasticidin for HEXIM1-transfected cells. Expression of POXDL and TRA-1-60 in the HEXIM1-transfected cells was determined by FACS analysis. (B) The TRA-1-60 negative and TRA-1-60 positive HEXIM1-transfected HES-3 cells (i.e. HEXIM1 Tra pos. and HEXIM1 Tra neg, respectively) were sorted and the expression of HEXIM1 in both populations was examined by QRT-PCR. Cells transfected with an empty vector were used as a control.

HES-3 cells transfected with an empty vector were used as the negative control. Cells used for the HEXIM1 overexpression assays were cultured in pluripotent conditions. Differentiation was assessed based on the expression of the pluripotent genes, PODXL and Tra-1-60, as determined by flow cytometry. In the control cells, about 10% of cells were observed to spontaneously differentiate (Figure 5A, Control). This was in contrast to 50% in the HEXIM-1-transfected HES-3 cells (Figure 5A, HEXIM1). Based on the expression of Tra-1-60, two distinct populations of HES-3 cells, Tra-negative and Tra-positive corresponding to differentiated and undifferentiated cells, were observed (Figure 5A). We sorted these two distinct groups and analyzed the expression of HEXIM1 by QRT-PCR. An 8-fold increase in the HEXIM1 mRNA level was detected in the Tra-negative HES-3 cells, compared to a 3-fold increase of HEXIM1 in the Tra-positive population (Figure 5B).

**Figure 6:**
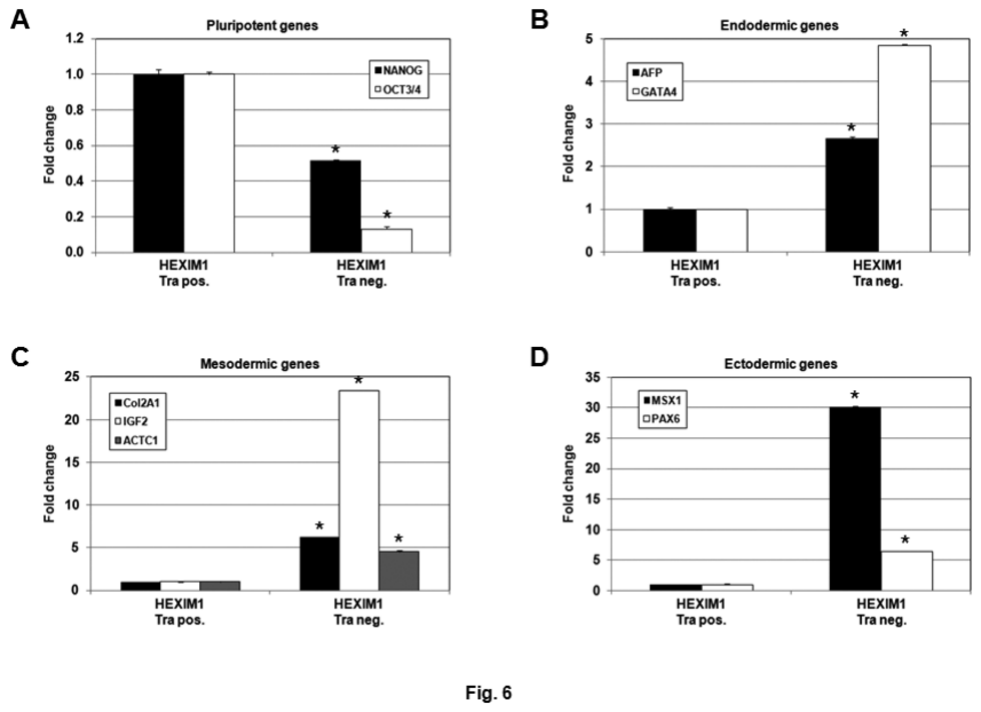
High-level expression of HEXIM1 up-regulates the marker genes of three germ layers in hPSCs. HES-3 cells were transiently transfected with a HEXIM1 expression plasmid, followed by antibiotic selection for the HEXIM1-transfected cells. The differentiated and undifferentiated populations of the HEXIM1-transfected HES-3 cells were sorted by FACS analysis using an anti-TRA-1-60 antibody. The mRNAs prepared from the TRA-1-60 positive and negative HEXIM1-transfected HES-3 cells (i.e. HEXIM1 Tra pos. and HEXIM1 Tra neg, respectively) were analyzed by QRT-PCR to determine the expression of (A) pluripotent (NANOG and OCT3/4), (B) endodermal (AFP and GATA4), (C) mesodermal (Col2A1, IGF2, and ACTC1), and (D) ectodermal genes (MSX1, PAX6, and SOX1). Results were reproducible, and data from one experiment are presented. Error bars indicate the standard deviations between triplicates. *P<0.05 versus control.

Next, we went on to determine the lineage commitment of differentiated HEXIM1-induced hPSC. Sorted Tra-negative and Tra-positive populations of the HEXIM1-transfected HES-3 cells were interrogated for expression of marker genes from the different germ layers. The expression patterns of HEXIM1 and pluripotent marker genes in the differentiated HEXIM1-overexpressing HES-3 cells were first confirmed by QRT-PCR (Figure 6A and data not shown). Up-regulation of the marker genes from all three germ layers was observed in the HEXIM1-transfected/Tra-negative HES-3, suggesting that there was no restriction to specific lineage commitment in HEXIM1-overexpressing cells (Figure 6B–D). This further demonstrated the importance of HEXIM1 in governing the regulatory mechanism of hPSC differentiation. Taken together, these results suggest that HEXIM1 may function as an inducer of hPSC differentiation when its expression surpasses a certain threshold level.

## Discussion

HEXIM1 functions as a P-TEFb inhibitor when associating with 7SK snRNA. The 7SK snRNA-bound HEXIM1 binds to P-TEFb through the interaction with cyclin T1, resulting in suppressing the kinase activity of P-TEFb [25,57]. In addition to cyclin T1, other HEXIM1 binding proteins have been reported [32–38]. We previously identified NPM, HDM2, and p53 as HEXIM1 binding proteins [34,35,39]. Overexpression of NPM led to proteasome-mediated degradation of HEXIM1. Furthermore, the cytoplasmic mutant of NPM, NPMc+, was found to associate with and sequester a portion of HEXIM1 in the cytoplasm and thus stimulated RNA Pol II transcription [34]. Significant lower protein levels of HEXIM1 and NPM were observed in the acute myeloid leukemia cell line with NPMc+ mutant [58]. We also showed that HDM2, a p53-specific E3 ubiquitin ligase, ubiquitinated HEXIM1. However, the HDM2-induced ubiquitination of HEXIM1 does not lead to proteasome-mediated protein degradation of HEXIM1, but enhances the inhibitory effects of HEXIM1 on the P-TEFb-dependent transcription [35]. Recently, we identified HEXIM1 as a positive regulator of p53. HEXIM1 inhibits ubiquitination and degradation of p53 mediated by HDM2, and therefore, enhances the protein stability of p53 [39].

HEXIM1 regulates the functions of its binding proteins through the P-TEFb-dependent and -independent mechanisms. Cyclin T1 and HEXIM1 are identified as the ERα binding proteins, and both proteins bind to the same activation domain of ERα [36,59]. Cyclin T1 functions as an activator of ERα, while the activity of ERα is inhibited when associated with HEXIM1 [36]. HEXIM1 may compete with cyclin T1 for binding to ERα, and therefore, the transcriptional activity of ERα is modulated by HEXIM1 through a P-TEFb-dependent mechanism [36]. In addition, HEXIM1 has been shown to negatively regulate the GR-mediated transcription through the protein–protein interaction with GR. However, HEXIM1 forms a distinct complex with GR in the absence of 7SK RNA, CDK9, and cyclin T1, indicating that the inhibition of GR by HEXIM1 is P-TEFb-independent [38].

Here we observed distinct expression patterns of CDK9, cyclin T1, and HEXIM1 proteins between pluripotent and differentiated hPSCs, suggesting a potential role of P-TEFb and/or HEXIM1 in the regulation of hPSCs (Figure 1). No sign of differentiation was detected when HES-3 cells were incubated with flavopiridol, a potent P-TEFb inhibiting compound (Figure 2A). Although the negative results were obtained, we still could not rule out the possible requirement of P-TEFb for hPSC differentiation. P-TEFb has been shown to regulate the transcription of most cellular genes, which include the ones required for hPSC pluripotency/differentiation as well as the fundamental cellular functions [18–22]. This could help to explain why severe cytotoxicity was detected when cell were incubated with high doses of flavopiridol. We later demonstrated that treatment of HMBA and overexpression of HEXIM1 induced the differentiation of hPSCs (Figures 3-6). Taken together, we conclude that the HEXIM1 mediates hPSC differentiation through a P-TEFb-independent pathway.

HMBA is a hybrid bipolar compound that induces terminal differentiation in transformed cells in culture [29,30]. We found that treatment of HMBA stimulated differentiation of multiple hPSC lines, accompanied with the increased levels of HEXIM1 (Figures 3 and S2). It was noted that hPSC differentiation was induced only when treated with high concentrations of HMBA (i.e. 5 or 10 mM) (Figure 3). Elevated protein levels of HEXIM1 were observed at lower amounts of HMBA (i.e. 1 or 3 mM). However, under these conditions, no significant changes in the pluripotent status of hPSCs were detected (Figure 3). This observation suggests that expression levels of HEXIM1 may need to surpass a certain “threshold” to initiate the differentiation of hPSCs. This hypothesis was supported by HEXIM1 overexpression assays. Compared to the control, ectopic expression of HEXIM1 significantly increased the population of differentiated HES-3 cells (Figure 5A). Notably, overexpression of HEXIM1 did not guarantee absolute commitment to differentiation: about half of the HEXIM1-transfected HES-3 cells remained undifferentiated (Figure 5A). Analysis of the differentiated and undifferentiated populations from the HEXIM1-transfected HES-3 cells revealed that differentiation corresponded to cells with high-level expression of HEXIM1 (Figure 5B, 8-fold); whilst the undifferentiated cells only had 3-fold up-regulation of HEXIM1 (Figure 5B).

We planned to investigate the impact of HEXIM1 knockdown on hPSC differentiation. Four short hairpin RNAs (shRNAs) against HEXIM1 were purchased. HEK293 cells were transfected with an individual HEXIM1 shRNA and the effectiveness of each shRNA was determined by western blot. One of the shRNAs showing consistent knockdown of HEXIM1 was selected for the knockdown experiment using HES-3 cells. Unfortunately, after 3 attempts, we were unable to obtain for a stable HEXIM1 knockdown cell line after antibiotic selection (data not shown). In spite of that, our results obtained from HMBA treatment (Figure 3) and HEXIM1 overexpression (Figures 5 and 6) assays suggest a role for HEXIM1 in the differentiation of hPSCs. As shown in Figures 3C and 5B, the protein level of HEXIM1 needs to surpass a certain “threshold” to initiate the differentiation. Therefore, it is expected that knockdown of HEXIM1 may further remain hPSCs in the pluripotent status.

Incubation of hPSCs with LY294002 and HMBA resulted in the differentiation of hPSCs (Figures 1 and 3). Previously, we demonstrated that the inhibition of PI3K/AKT pathway in hPSCs resulted in down-regulation of pluripotent markers, including OCT3/4, PODXL, and Tra-1-60 [43]. HMBA had also been shown to have effects in modulating the PI3K/AKT pathway, both as an inhibitor and an activator of the signaling pathway [60,61]. Up-regulation of HEXIM1 expression was found in the hPSCs treated with LY294002 and HMBA (Figures 1 and 3), and furthermore, overexpression of HEXIM1 resulted in hPSC differentiation (Figures 5 and 6). Collectively, our findings suggest the potential involvement of HEXIM1 as well as the PI3K/AKT pathway in the regulation of hPSC differentiation.

Lineage commitment of the hPSC differentiation induced by HMBA treatment and HEXIM1 overexpression was examined. Incubation of HMBA resulted in differentiation of hPSCs towards the mesoderm and ectoderm lineages (Figure 4). However, ectopic expression of HEXIM1 failed to show any lineage-specific restriction but instead activated all the marker genes from three germ layers examined (Figure 6). Incubation of HMBA could have a profound effect on hPSCs by influencing multiple cellular molecules and signaling pathways. Induction of HEXIM1 expression could be only one of several cellular responses caused by the compound. For example, HMBA is also known to modulate the MAPK signaling pathway and NF-κB activation [34] apart from PI3K/AKT as discussed previously. Therefore, it is not surprising to observe different phenotypes caused by HMBA incubation and HEXIM1 overexpression. It is possible that HEXIM1 may be involved in the early initiating stage of hPSC differentiation but not required for any lineage- or tissue-specific commitment in the later stages. Nevertheless, this is the first demonstration of the role of HEXIM1 in hPSCs and future work is required to investigate the molecular mechanism of hPSC differentiation mediated by HEXIM1 in detail.

## Supporting Information

Table S1 Primers used for qRT-PCR.
(DOCX)

Figure S1 **Treatment of HMBA resulted in morphological changes in hESC.** HES-3 cells were incubated with 1, 3, 5, or 10 mM HMBA for 7 PDs. Cells with higher concentrations of HMBA displayed more differentiated morphology when compared to vehicle control. The cystic-like areas are pointed by red arrows.
(DOCX)

Figure S2 **Treatment of HMBA leads to differentiation of HES-2 and iPS cells.** (A) HES-2 and iPS cells were incubated with 5 or 10 mM HMBA for 7 PDs, followed by FACS analysis. Percentages of cells expressing OCT3/4, PODXL, and Tra-1-60, were indicated (i.e. open histograms). Cells treated with 1% ethanol were used as the vehicle control. (B) Expression of HEXIM1 and OCT4 in the HMBA-treated HES-2 and iPS cells were examined by western blotting. Actin was used as a loading control.
(DOCX)

Figure S3 **HES-3 cells were treated with 20 µM LY294002 for 7 PDs.** 0.2% DMSO was used as vehicle control. The mRNAs prepared from the treated HES-3 cells were analyzed by QRT-PCR to determine the expression of (A) pluripotent (OCT3/4 and NANOG), (B) endodermal (GATA4 and AFP), (C) mesodermal (Col2A1, IGF2, and ACTC1), and (D) ectodermal genes (MSX1, PAX6, and SOX1).
(DOCX)

## References

[1] Reubinoff BE, Pera MF, Fong CY, Trounson A, Bongso A (2000) Embryonic stem cell lines from human blastocysts: somatic differentiation in vitro. Nat Biotechnol 18: 399-404. doi:10.1038/74447. PubMed: 10748519. 10748519 10.1038/74447

[2] Thomson JA, Itskovitz-Eldor J, Shapiro SS, Waknitz MA, Swiergiel JJ et al. (1998) Embryonic stem cell lines derived from human blastocysts. Science 282: 1145-1147. doi:10.1126/science.282.5391.1145. PubMed: 9804556. 9804556 10.1126/science.282.5391.1145

[3] Takahashi K, Tanabe K, Ohnuki M, Narita M, Ichisaka T et al. (2007) Induction of pluripotent stem cells from adult human fibroblasts by defined factors. Cell 131: 861-872. doi:10.1016/j.cell.2007.11.019. PubMed: 18035408. 18035408 10.1016/j.cell.2007.11.019

[4] Yu J, Vodyanik MA, Smuga-Otto K, Antosiewicz-Bourget J, Frane JL et al. (2007) Induced pluripotent stem cell lines derived from human somatic cells. Science 318: 1917-1920. doi:10.1126/science.1151526. PubMed: 18029452. 18029452 10.1126/science.1151526

[5] He JQ, Ma Y, Lee Y, Thomson JA, Kamp TJ (2003) Human embryonic stem cells develop into multiple types of cardiac myocytes: action potential characterization. Circ Res 93: 32-39. doi:10.1161/01.RES.0000080317.92718.99. PubMed: 12791707. 12791707 10.1161/01.RES.0000080317.92718.99

[6] Snir M, Kehat I, Gepstein A, Coleman R, Itskovitz-Eldor J et al. (2003) Assessment of the ultrastructural and proliferative properties of human embryonic stem cell-derived cardiomyocytes. Am J Physiol Heart Circ Physiol 285: H2355-H2363. PubMed: 14613910. 14613910 10.1152/ajpheart.00020.2003

[7] Gherghiceanu M, Barad L, Novak A, Reiter I, Itskovitz-Eldor J et al. (2011) Cardiomyocytes derived from human embryonic and induced pluripotent stem cells: comparative ultrastructure. J Cell Mol Med 15: 2539-2551. doi:10.1111/j.1582-4934.2011.01417.x. PubMed: 21883888. 21883888 10.1111/j.1582-4934.2011.01417.x PMC3822963

[8] Assady S, Maor G, Amit M, Itskovitz-Eldor J, Skorecki KL et al. (2001) Insulin production by human embryonic stem cells. Diabetes 50: 1691-1697. doi:10.2337/diabetes.50.8.1691. PubMed: 11473026. 11473026 10.2337/diabetes.50.8.1691

[9] D’Amour KA, Bang AG, Eliazer S, Kelly OG, Agulnick AD et al. (2006) Production of pancreatic hormone-expressing endocrine cells from human embryonic stem cells. Nat Biotechnol 24: 1392-1401. doi:10.1038/nbt1259. PubMed: 17053790. 17053790 10.1038/nbt1259

[10] Reubinoff BE, Itsykson P, Turetsky T, Pera MF, Reinhartz E et al. (2001) Neural progenitors from human embryonic stem cells. Nat Biotechnol 19: 1134-1140. doi:10.1038/nbt1201-1134. PubMed: 11731782. 11731782 10.1038/nbt1201-1134

[11] Pankratz MT, Li XJ, Lavaute TM, Lyons EA, Chen X et al. (2007) Directed neural differentiation of human embryonic stem cells via an obligated primitive anterior stage. Stem Cells 25: 1511-1520. doi:10.1634/stemcells.2006-0707. PubMed: 17332508. 17332508 10.1634/stemcells.2006-0707 PMC2743478

[12] Song B, Sun G, Herszfeld D, Sylvain A, Campanale NV et al. (2012) Neural differentiation of patient specific iPS cells as a novel approach to study the pathophysiology of multiple sclerosis. Stem Cell Res 8: 259-273. doi:10.1016/j.scr.2011.12.001. PubMed: 22265745. 22265745 10.1016/j.scr.2011.12.001

[13] Peng J, Zhu Y, Milton JT, Price DH (1998) Identification of multiple cyclin subunits of human P-TEFb. Genes Dev 12: 755-762. doi:10.1101/gad.12.5.755. PubMed: 9499409. 9499409 10.1101/gad.12.5.755 PMC316581

[14] Graña X, De Luca A, Sang N, Fu Y, Claudio PP et al. (1994) PITALRE, a nuclear CDC2-related protein kinase that phosphorylates the retinoblastoma protein in vitro. Proc Natl Acad Sci U S A 91: 3834-3838. doi:10.1073/pnas.91.9.3834. PubMed: 8170997. 8170997 10.1073/pnas.91.9.3834 PMC43676

[15] Fu TJ, Peng J, Lee G, Price DH, Flores O (1999) Cyclin K functions as a CDK9 regulatory subunit and participates in RNA polymerase II transcription. J Biol Chem 274: 34527-34530. doi:10.1074/jbc.274.49.34527. PubMed: 10574912. 10574912 10.1074/jbc.274.49.34527

[16] Chao SH, Fujinaga K, Marion JE, Taube R, Sausville EA et al. (2000) Flavopiridol inhibits P-TEFb and blocks HIV-1 replication. J Biol Chem 275: 28345-28348. doi:10.1074/jbc.C000446200. PubMed: 10906320. 10906320 10.1074/jbc.C000446200

[17] Peterlin BM, Price DH (2006) Controlling the elongation phase of transcription with P-TEFb. Mol Cell 23: 297-305. doi:10.1016/j.molcel.2006.06.014. PubMed: 16885020. 16885020 10.1016/j.molcel.2006.06.014

[18] Chao SH, Price DH (2001) Flavopiridol inactivates P-TEFb and blocks most RNA polymerase II transcription in vivo. J Biol Chem 276: 31793-31799. doi:10.1074/jbc.M102306200. PubMed: 11431468. 11431468 10.1074/jbc.M102306200

[19] Blagosklonny MV (2004) Flavopiridol, an inhibitor of transcription: implications, problems and solutions. Cell Cycle 3: 1537-1542. doi:10.4161/cc.3.12.1278. PubMed: 15539947. 15539947 10.4161/cc.3.12.1278

[20] Guenther MG, Levine SS, Boyer LA, Jaenisch R, Young RA (2007) A chromatin landmark and transcription initiation at most promoters in human cells. Cell 130: 77-88. doi:10.1016/j.cell.2007.05.042. PubMed: 17632057. 17632057 10.1016/j.cell.2007.05.042 PMC3200295

[21] Zeitlinger J, Stark A, Kellis M, Hong JW, Nechaev S et al. (2007) RNA polymerase stalling at developmental control genes in the Drosophila melanogaster embryo. Nat Genet 39: 1512-1516. doi:10.1038/ng.2007.26. PubMed: 17994019. 17994019 10.1038/ng.2007.26 PMC2824921

[22] Muse GW, Gilchrist DA, Nechaev S, Shah R, Parker JS et al. (2007) RNA polymerase is poised for activation across the genome. Nat Genet 39: 1507-1511. doi:10.1038/ng.2007.21. PubMed: 17994021. 17994021 10.1038/ng.2007.21 PMC2365887

[23] Price DH (2008) Poised polymerases: on your mark... get set... go! Mol Cell 30(1): 7-10. doi:10.1016/j.molcel.2008.03.001. PubMed: 18406322. 18406322 10.1016/j.molcel.2008.03.001

[24] Nguyen VT, Kiss T, Michels AA, Bensaude O (2001) 7SK small nuclear RNA binds to and inhibits the activity of CDK9/cyclin T complexes. Nature 414: 322-325. doi:10.1038/35104581. PubMed: 11713533. 11713533 10.1038/35104581

[25] Michels AA, Nguyen VT, Fraldi A, Labas V, Edwards M et al. (2003) MAQ1 and 7SK RNA interact with CDK9/cyclin T complexes in a transcription-dependent manner. Mol Cell Biol 23: 4859-4869. doi:10.1128/MCB.23.14.4859-4869.2003. PubMed: 12832472. 12832472 10.1128/MCB.23.14.4859-4869.2003 PMC162212

[26] Yang Z, Zhu Q, Luo K, Zhou Q (2001) The 7SK small nuclear RNA inhibits the CDK9/cyclin T1 kinase to control transcription. Nature 414: 317-322. doi:10.1038/35104575. PubMed: 11713532. 11713532 10.1038/35104575

[27] Yik JH, Chen R, Nishimura R, Jennings JL, Link AJ et al. (2003) Inhibition of P-TEFb (CDK9/Cyclin T) kinase and RNA polymerase II transcription by the coordinated actions of HEXIM1 and 7SK snRNA. Mol Cell 12: 971-982. doi:10.1016/S1097-2765(03)00388-5. PubMed: 14580347. 14580347 10.1016/s1097-2765(03)00388-5

[28] Kusuhara M, Nagasaki K, Kimura K, Maass N, Manabe T et al. (1999) Cloning of hexamethylene-bis-acetamide-inducible transcript, HEXIM1, in human vascular smooth muscle cells. Biomed Res 20: 273-279.

[29] Fibach E, Reuben RC, Rifkind RA, Marks PA (1977) Effect of hexamethylene bisacetamide on the commitment to differentiation of murine erythroleukemia cells. Cancer Res 37: 440-444. PubMed: 264411. 264411

[30] Marks PA, Richon VM, Kiyokawa H, Rifkind RA (1994) Inducing differentiation of transformed cells with hybrid polar compounds: a cell cycle-dependent process. Proc Natl Acad Sci U S A 91: 10251-10254. doi:10.1073/pnas.91.22.10251. PubMed: 7937935. 7937935 10.1073/pnas.91.22.10251 PMC44997

[31] Dey A, Chao SH, Lane DP (2007) HEXIM1 and the control of transcription elongation: from cancer and inflammation to AIDS and cardiac hypertrophy. Cell Cycle 6: 1856-1863. doi:10.4161/cc.6.15.4556. PubMed: 17671421. 17671421 10.4161/cc.6.15.4556

[32] Galatioto J, Mascareno E, Siddiqui MA (2010) CLP-1 associates with MyoD and HDAC to restore skeletal muscle cell regeneration. J Cell Sci 123: 3789-3795. doi:10.1242/jcs.073387. PubMed: 20940258. 20940258 10.1242/jcs.073387 PMC2964110

[33] Czudnochowski N, Vollmuth F, Baumann S, Vogel-Bachmayr K, Geyer M (2010) Specificity of Hexim1 and Hexim2 complex formation with cyclin T1/T2, importin alpha and 7SK snRNA. J Mol Biol 395: 28-41. doi:10.1016/j.jmb.2009.10.055. PubMed: 19883659. 19883659 10.1016/j.jmb.2009.10.055

[34] Gurumurthy M, Tan CH, Ng R, Zeiger L, Lau J et al. (2008) Nucleophosmin interacts with HEXIM1 and regulates RNA polymerase II transcription. J Mol Biol 378: 302-317. doi:10.1016/j.jmb.2008.02.055. PubMed: 18371977. 18371977 10.1016/j.jmb.2008.02.055

[35] Lau J, Lew QJ, Diribarne G, Michels AA, Dey A et al. (2009) Ubiquitination of HEXIM1 by HDM2. Cell Cycle 8: 2247-2254. doi:10.4161/cc.8.14.9015. PubMed: 19617712. 19617712 10.4161/cc.8.14.9015

[36] Wittmann BM, Fujinaga K, Deng H, Ogba N, Montano MM (2005) The breast cell growth inhibitor, estrogen down regulated gene 1, modulates a novel functional interaction between estrogen receptor alpha and transcriptional elongation factor cyclin T1. Oncogene 24: 5576-5588. doi:10.1038/sj.onc.1208728. PubMed: 15940264. 15940264 10.1038/sj.onc.1208728

[37] Ouchida R, Kusuhara M, Shimizu N, Hisada T, Makino Y et al. (2003) Suppression of NF-kappaB-dependent gene expression by a hexamethylene bisacetamide-inducible protein HEXIM1 in human vascular smooth muscle cells. Genes Cells 8: 95-107. doi:10.1046/j.1365-2443.2003.00618.x. PubMed: 12581153. 12581153 10.1046/j.1365-2443.2003.00618.x

[38] Shimizu N, Ouchida R, Yoshikawa N, Hisada T, Watanabe H et al. (2005) HEXIM1 forms a transcriptionally abortive complex with glucocorticoid receptor without involving 7SK RNA and positive transcription elongation factor b. Proc Natl Acad Sci U S A 102: 8555-8560. doi:10.1073/pnas.0409863102. PubMed: 15941832. 15941832 10.1073/pnas.0409863102 PMC1150813

[39] Lew QJ, Chia YL, Chu KL, Lam YT, Gurumurthy M et al. (2012) Identification of HEXIM1 as a positive regulator of p53. J Biol Chem 287: 36443-36454. doi:10.1074/jbc.M112.374157. PubMed: 22948151. 22948151 10.1074/jbc.M112.374157 PMC3476310

[40] Choo A, Padmanabhan J, Chin A, Fong WJ, Oh SK (2006) Immortalized feeders for the scale-up of human embryonic stem cells in feeder and feeder-free conditions. J Biotechnol 122: 130-141. doi:10.1016/j.jbiotec.2005.09.008. PubMed: 16233925. 16233925 10.1016/j.jbiotec.2005.09.008

[41] Ding V, Choo AB, Oh SK (2006) Deciphering the importance of three key media components in human embryonic stem cell cultures. Biotechnol Lett 28: 491-495. doi:10.1007/s10529-006-0005-8. PubMed: 16614931. 16614931 10.1007/s10529-006-0005-8

[42] Choo AB, Tan HL, Ang SN, Fong WJ, Chin A et al. (2008) Selection against undifferentiated human embryonic stem cells by a cytotoxic antibody recognizing podocalyxin-like protein-1. Stem Cells 26: 1454-1463. doi:10.1634/stemcells.2007-0576. PubMed: 18356574. 18356574 10.1634/stemcells.2007-0576

[43] Ding VM, Ling L, Natarajan S, Yap MG, Cool SM et al. (2010) FGF-2 modulates Wnt signaling in undifferentiated hESC and iPS cells through activated PI3-K/GSK3beta signaling. J Cell Physiol 225: 417-428. doi:10.1002/jcp.22214. PubMed: 20506199. 20506199 10.1002/jcp.22214

[44] Chan KK, Wu SM, Nissom PM, Oh SK, Choo AB (2008) Generation of high-level stable transgene expressing human embryonic stem cell lines using Chinese hamster elongation factor-1 alpha promoter system. Stem Cells Dev 17: 825-836. doi:10.1089/scd.2007.0233. PubMed: 18788934. 18788934 10.1089/scd.2007.0233

[45] Livak KJ, Schmittgen TD (2001) Analysis of relative gene expression data using real-time quantitative PCR and the 2(-Delta Delta C(T)) Method. Methods 25(4): 402-408. doi:10.1006/meth.2001.1262. PubMed: 11846609. 11846609 10.1006/meth.2001.1262

[46] Paling NR, Wheadon H, Bone HK, Welham MJ (2004) Regulation of embryonic stem cell self-renewal by phosphoinositide 3-kinase-dependent signaling. J Biol Chem 279: 48063-48070. doi:10.1074/jbc.M406467200. PubMed: 15328362. 15328362 10.1074/jbc.M406467200

[47] Nichols J, Zevnik B, Anastassiadis K, Niwa H, Klewe-Nebenius D et al. (1998) Formation of pluripotent stem cells in the mammalian embryo depends on the POU transcription factor Oct4. Cell 95: 379-391. doi:10.1016/S0092-8674(00)81769-9. PubMed: 9814708. 9814708 10.1016/s0092-8674(00)81769-9

[48] Chao SH, Walker JR, Chanda SK, Gray NS, Caldwell JS (2003) Identification of homeodomain proteins, PBX1 and PREP1, involved in the transcription of murine leukemia virus. Mol Cell Biol 23: 831-841. doi:10.1128/MCB.23.3.831-841.2003. PubMed: 12529389. 12529389 10.1128/MCB.23.3.831-841.2003 PMC140703

[49] Dziadek M, Adamson E (1978) Localization and synthesis of alphafoetoprotein in post-implantation mouse embryos. J Embryol Exp Morphol 43: 289-313. PubMed: 75937. 75937

[50] Arceci RJ, King AA, Simon MC, Orkin SH, Wilson DB (1993) Mouse GATA-4: a retinoic acid-inducible GATA-binding transcription factor expressed in endodermally derived tissues and heart. Mol Cell Biol 13: 2235-2246. PubMed: 8455608. 8455608 10.1128/mcb.13.4.2235-2246.1993 PMC359544

[51] Yan YL, Hatta K, Riggleman B, Postlethwait JH (1995) Expression of a type II collagen gene in the zebrafish embryonic axis. Dev Dyn 203: 363-376. doi:10.1002/aja.1002030308. PubMed: 8589433. 8589433 10.1002/aja.1002030308

[52] Morali OG, Jouneau A, McLaughlin KJ, Thiery JP, Larue L (2000) IGF-II promotes mesoderm formation. Dev Biol 227: 133-145. doi:10.1006/dbio.2000.9875. PubMed: 11076682. 11076682 10.1006/dbio.2000.9875

[53] Zhang SX, Garcia-Gras E, Wycuff DR, Marriot SJ, Kadeer N et al. (2005) Identification of direct serum-response factor gene targets during Me2SO-induced P19 cardiac cell differentiation. J Biol Chem 280: 19115-19126. doi:10.1074/jbc.M413793200. PubMed: 15699019. 15699019 10.1074/jbc.M413793200

[54] Coelho CN, Upholt WB, Kosher RA (1993) The expression pattern of the chicken homeobox-containing gene GHox-7 in developing polydactylous limb buds suggests its involvement in apical ectodermal ridge-directed outgrowth of limb mesoderm and in programmed cell death. Differentiation 52: 129-137. doi:10.1111/j.1432-0436.1993.tb00623.x. PubMed: 8097171. 8097171 10.1111/j.1432-0436.1993.tb00623.x

[55] Li HS, Yang JM, Jacobson RD, Pasko D, Sundin O (1994) Pax-6 is first expressed in a region of ectoderm anterior to the early neural plate: implications for stepwise determination of the lens. Dev Biol 162: 181-194. doi:10.1006/dbio.1994.1077. PubMed: 8125186. 8125186 10.1006/dbio.1994.1077

[56] Pevny LH, Sockanathan S, Placzek M, Lovell-Badge R (1998) A role for SOX1 in neural determination. Development 125: 1967-1978. PubMed: 9550729. 9550729 10.1242/dev.125.10.1967

[57] Schulte A, Czudnochowski N, Barboric M, Schönichen A, Blazek D et al. (2005) Identification of a cyclin T-binding domain in Hexim1 and biochemical analysis of its binding competition with HIV-1 Tat. J Biol Chem 280: 24968-24977. doi:10.1074/jbc.M501431200. PubMed: 15855166. 15855166 10.1074/jbc.M501431200

[58] Lew QJ, Tan CH, Gurumurthy M, Chu KL, Cheong N et al. (2011) NPMc(+) AML cell line shows differential protein expression and lower sensitivity to DNA-damaging and p53-inducing anticancer compounds. Cell Cycle 10: 1978-1987. doi:10.4161/cc.10.12.15859. PubMed: 21558800. 21558800 10.4161/cc.10.12.15859

[59] Wittmann BM, Wang N, Montano MM (2003) Identification of a novel inhibitor of breast cell growth that is down-regulated by estrogens and decreased in breast tumors. Cancer Res 63: 5151-5158. PubMed: 12941847. 12941847

[60] Contreras X, Barboric M, Lenasi T, Peterlin BM (2007) HMBA releases P-TEFb from HEXIM1 and 7SK snRNA via PI3K/Akt and activates HIV transcription. PLOS Pathog 3: 1459-1469. PubMed: 17937499. 17937499 10.1371/journal.ppat.0030146 PMC2014796

[61] Dey A, Wong E, Kua N, Teo HL, Tergaonkar V et al. (2008) Hexamethylene bisacetamide (HMBA) simultaneously targets AKT and MAPK pathway and represses NF kappaB activity: implications for cancer therapy. Cell Cycle 7: 3759-3767. doi:10.4161/cc.7.23.7213. PubMed: 19029824. 19029824 10.4161/cc.7.23.7213

