# Supplementary material for: HEXIM1 Induces Differentiation of Human Pluripotent Stem Cells
Source: PLoS One. 2013 Aug 20;8(8):e72823. doi: 10.1371/journal.pone.0072823 (PMC3748041; doi:10.1371/journal.pone.0072823)
Supplement: Table S1 — Primers used for qRT-PCR. (DOCX) [file pone.0072823.s001.docx]

**Table S1: Primers used for qRT-PCR**

| **Gene** | **Primer Sequence** |
| --- | --- |
| **AFP** | F: TCCCTCCTGCATTCTCTGATG  R: CCTGAGCTTGGCACAGATCC |
| **ACTC** | F: ATTG GCAATGAGCGCTTCC  R: TGCCAGCAGATTCCATACCA |
| **COL2A** | F: GCCATGAAGGTTTTCTGCAAC  R: TTGGGAACGTTTGCTGGATT |
| **GATA4** | F: ACAGACCAGCTCCAAGCAGG  R: CGTGACTGTCGGCCAAGAC |
| **IGF2** | F: CATCTCCCTTCTCACGGGAAT  R: GTTGCTATTTTCGGATGGCC |
| **MSX1** | F: GCCATGTCTCCTGCATAGCTT  R: CGCTTTTCTTGCCTGGTGTC |
| **Nanog** | F: GAAAAACAACTGGCCGAAGAAT  R: GGTGCTGAGGCCTTCTGC |
| **OCT4** | F: AAACCCGGAGGAGTCCCAG  R: TGGCAAATTGCTCGAGTTCTT |
| **PAX6** | F: CCAGCTTCACCATGGCAAAT  R: GGCAGCATGCAGGAGTATGAG |
| **SOX1** | F: CACAACTCGGAGATCAGCAA  R: GTCCTTCTTGAGCAGCGTCT |
| **HEXIM1** | F: CGAGGAGGACAGTAGGTGG  R: CAGGCAGCTAGATTCTGGACA |
| **18S** | F: CGCCGCGCTCTACCTTACCTA  R: TAGGAGAGGAGCGAGCGACCA |
| **GAPDH** | F: AACAGCCTCAAGATCATCAGC  R: GGATGATGTTCTGGAGAGCC |
