# Supplementary material for: HEXIM1 Induces Differentiation of Human Pluripotent Stem Cells
Source: PLoS One. 2013 Aug 20;8(8):e72823. doi: 10.1371/journal.pone.0072823 (PMC3748041; doi:10.1371/journal.pone.0072823)

**Figure S1.** Treatment of HMBA resulted in morphological changes in hESC. HES-3 cells were incubated with 1, 3, 5, or 10 mM HMBA for 7 PDs. Cells with higher concentrations of HMBA displayed more differentiated morphology when compared to vehicle control. The cystic-like areas are pointed by red arrows.


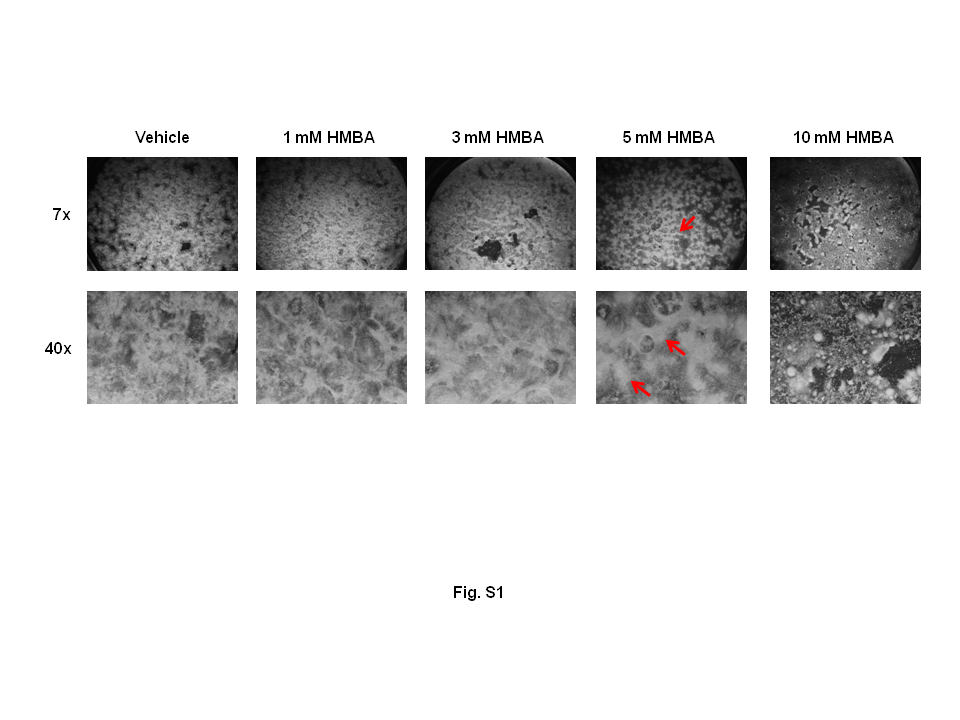

Supplement: Figure S1 — Treatment of HMBA resulted in morphological changes in hESC. HES-3 cells were incubated with 1, 3, 5, or 10 mM HMBA for 7 PDs. Cells with higher concentrations of HMBA displayed more differentiated morphology when compared to vehicle control. The cystic-like areas are pointed by red arrows. (DOCX) [file pone.0072823.s002.docx]
