# Supplementary material for: HEXIM1 Induces Differentiation of Human Pluripotent Stem Cells
Source: PLoS One. 2013 Aug 20;8(8):e72823. doi: 10.1371/journal.pone.0072823 (PMC3748041; doi:10.1371/journal.pone.0072823)

**Figure S2.** Treatment of HMBA leads to differentiation of HES-2 and iPS cells. (A) HES-2 and iPS cells were incubated with 5 or 10 mM HMBA for 7 PDs, followed by FACS analysis. Percentages of cells expressing OCT3/4, PODXL, and Tra-1-60, were indicated (i.e. open histograms). Cells treated with 1% ethanol were used as the vehicle control. (B) Expression of HEXIM1 and OCT4 in the HMBA-treated HES-2 and iPS cells were examined by western blotting. Actin was used as a loading control.


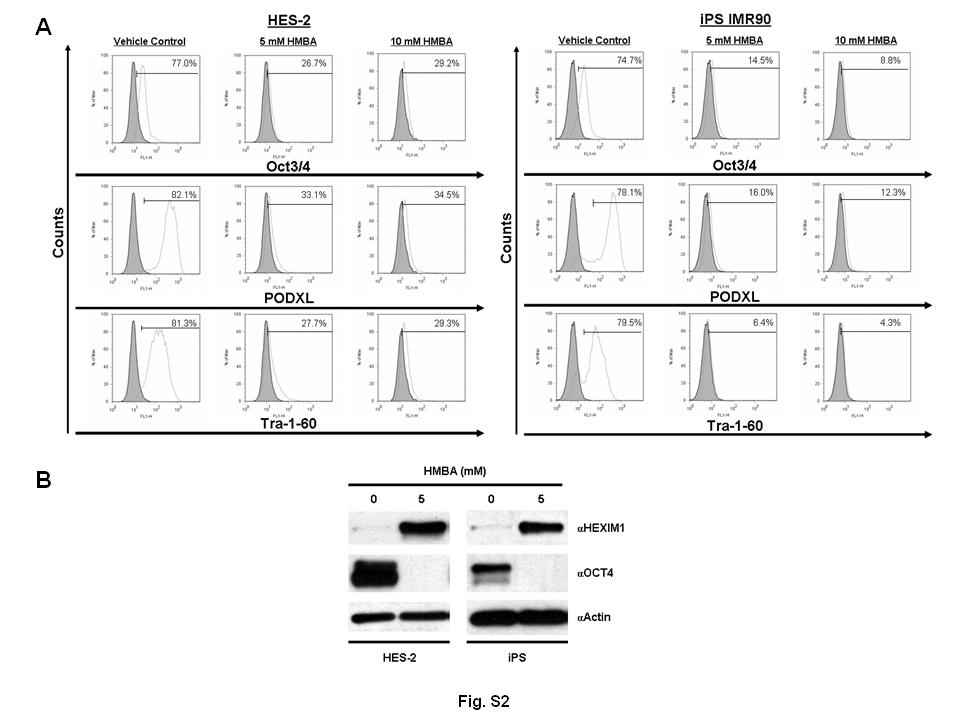

Supplement: Figure S2 — Treatment of HMBA leads to differentiation of HES-2 and iPS cells. (A) HES-2 and iPS cells were incubated with 5 or 10 mM HMBA for 7 PDs, followed by FACS analysis. Percentages of cells expressing OCT3/4, PODXL, and Tra-1-60, were indicated (i.e. open histograms). Cells treated with 1% ethanol were used as the vehicle control. (B) Expression of HEXIM1 and OCT4 in the HMBA-treated HES-2 and iPS cells were examined by western blotting. Actin was used as a loading control. (DOCX) [file pone.0072823.s003.docx]
