# Supplementary material for: HEXIM1 Induces Differentiation of Human Pluripotent Stem Cells
Source: PLoS One. 2013 Aug 20;8(8):e72823. doi: 10.1371/journal.pone.0072823 (PMC3748041; doi:10.1371/journal.pone.0072823)

**Figure S3.** HES-3 cells were treated with 20 µM LY294002 for 7 PDs. 0.2% DMSO was used as vehicle control. The mRNAs prepared from the treated HES-3 cells were analyzed by QRT-PCR to determine the expression of (A) pluripotent (OCT3/4 and NANOG), (B) endodermal (GATA4 and AFP), (C) mesodermal (Col2A1, IGF2, and ACTC1), and (D) ectodermal genes (MSX1, PAX6, and SOX1).


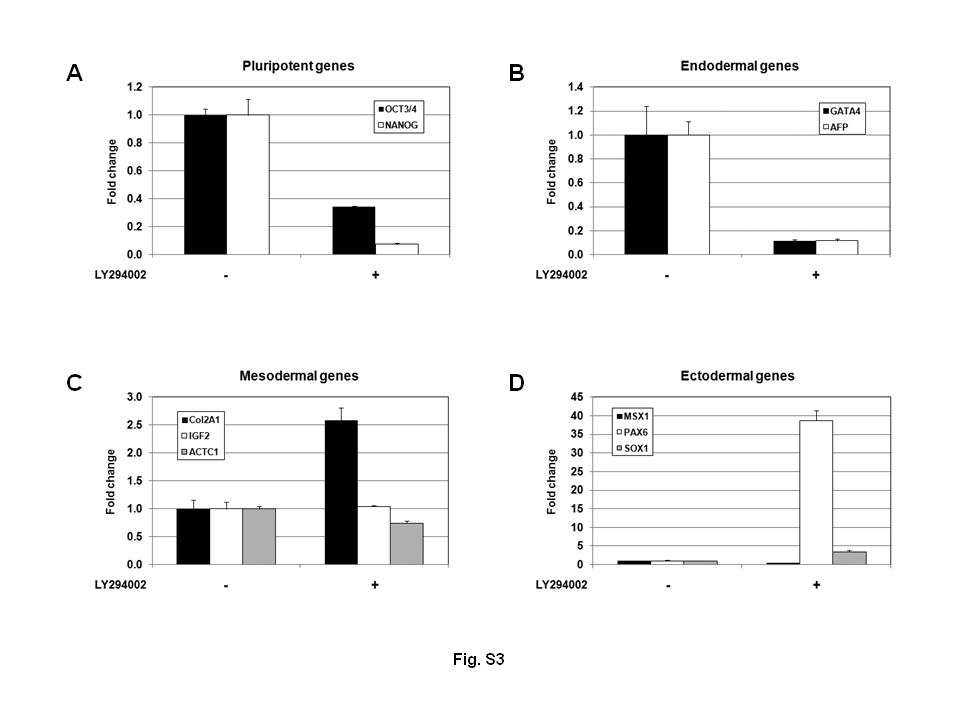

Supplement: Figure S3 — HES-3 cells were treated with 20 µM LY294002 for 7 PDs. 0.2% DMSO was used as vehicle control. The mRNAs prepared from the treated HES-3 cells were analyzed by QRT-PCR to determine the expression of (A) pluripotent (OCT3/4 and NANOG), (B) endodermal (GATA4 and AFP), (C) mesodermal (Col2A1, IGF2, and ACTC1), and (D) ectodermal genes (MSX1, PAX6, and SOX1). (DOCX) [file pone.0072823.s004.docx]
